# Identification of a hot-spot to enhance *Candida rugosa* lipase thermostability by rational design methods†

**DOI:** 10.1039/c7ra11679a

**Published:** 2018-01-09

**Authors:** Guanlin Li, Yuan Chen, Xingrong Fang, Feng Su, Li Xu, Yunjun Yan

**Affiliations:** Key Laboratory of Molecular Biophysics, The Ministry of Education, College of Life Science and Technology, Huazhong University of Science and Technology Wuhan 430074 P. R. China xuli@hust.edu.cn yanyunjun@hust.edu.cn +86-27-87792213 +86-27-87792213

## Abstract

Lipase is one of the most widely used classes of enzymes in biotechnological applications and organic chemistry. *Candida rugosa* lipases (CRL) can catalyze hydrolysis, esterification and transesterification with high regio-, stereo- and enantio-selectivity. However, thermal inactivation above 45 °C limits CRL's applications. Studies on improving the thermal stability of CRL are often limited by its slow-growing eukaryotic expression host, which is not suitable for large-scale screening. Identification of thermally stable mutants by rational design, regarded as an efficient substitution of experimental efforts, would provide a method for site-directed improvement of CRL. In this study, mutation-induced stability changes in CRL Lip1 were predicted by three rational design methods. Followed by conservative analyses and functional region exclusion, five mutants of a hot-spot, Asp457Phe, Asp457Trp, Asp457Met, Asp457Leu, and Asp457Tyr, were identified and prepared for enzymatic characterization. These five mutants increased the apparent melting temperature of Lip1 from 7.4 °C to 9.3 °C, with the most thermostable mutant, Asp457Phe, exhibiting a 5.5-fold longer half-life at 50 °C and a 10 °C increase in optimum temperature. Furthermore, pH stability of Lip1 was also enhanced due to the introduction of Asp457Phe mutation. The study demonstrates that thermally stable mutants of CRL could be identified with limited experimental efforts using rational design methods.

## Abstract 2

The thermostability of *Candida rugosa* lipase expressed in a eukaryotic host is enhanced with limited experimental effort based on rational design methods.

## Graphical abstract

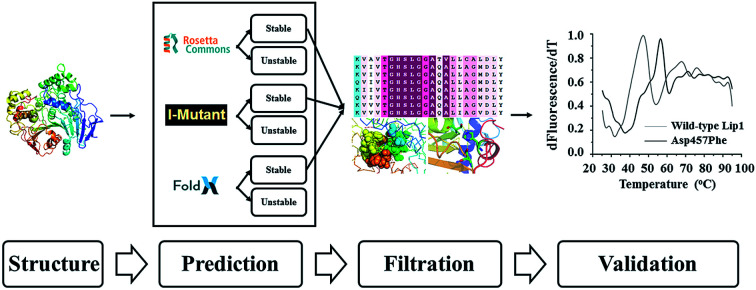

## Introduction

*Candida rugosa* (formerly *Candida cylindracea*) is a non-sporogenic, pseudofilamentous, unicellular, and non-pathogenic yeast which is generally regarded as safe.^1^ Lipases from *C. rugosa* (CRLs) are well-known lipases that have unparalleled broad specificity (regio-, and stereo-specificity) compared to others. They are non-specific to the ester bonds in glyceride or primary and secondary esters, and accept ester bonds formed with different chain-lengths of acid and alcohol moieties.^1^ Therefore, CRLs are a group of the most versatile and widely used enzymes for the catalytic decomposition and synthesis of ester compounds, as well as regioselective and chemoselective acylations and deacylations, in aqueous and organic media for industrial applications, including pharmaceuticals, food and cosmetics.^2^

*C. rugosa* synthesizes and secretes a mixture of lipase isoenzymes. It is well established that at least eight genes are involved in the *C. rugosa* lipase-producing machinery, six of which (Lip1-Lip5 and LipJ08) have been completely biochemically characterized.^3^ These isoenzymes differ in terms of amino acid sequence, isoelectric point and glycosylation degree.^4^ Among the eight isoforms, Lipl has the highest level of constitutive expression (more than 60% in crude commercial enzymes).^5^ Previously, heterologously overexpression of Lip1 in a *Pichia pastoris* system was successfully achieved in our laboratory.^6^

However, many enzymes derived from mesophilic microorganisms are unstable at high temperatures, and Lip1 is no exception. Lip1 is a mesophilic enzyme with an optimum temperature of 40 °C, starts inactivation at 45 °C, has a half-life of less than 1 hour at 50 °C, and loses nearly all catalytic activity at 60 °C.^7,8^ Poor thermal stability prevents Lip1 from being applied in industrial processes where relatively high temperatures are required.^9^ Literature surveys reveal that enhancing thermal stability of CRL *via* protein engineering is rarely reported. Recent strategies to stabilize Lip1 have focused on residues at the active center. Multiple sequence alignments and structural analyses resulted in acyl-binding residue Gly414 as a hotspot to increase thermostability.^9^ Based on B-factor ranking within 10 Å of the catalytic residues, high-throughput screening and ordered recombination mutagenesis, Phe344Ile/Phe434Tyr/Phe133Tyr/Phe121Tyr was also identified to enhance Lip1 stability.^10^ However, these strategies, stabilizing residues near the active center, are usually accompanied by loss of catalytic activity and alterations in substrate specificity.^11^ Furthermore, enhancing the thermostability of CRL is a challenging field since eukaryotic hosts have a long culture time and are not suited for high-throughput screening of thermostable mutants.^12^

So far, a variety of studies were carried out to improve the thermostability of enzymes, such as immobilization,^13,14^ medium engineering,^15^ and protein engineering.^16^ In term of protein engineering, irrational design, semi-rational design and rational design were three general categories employed to get thermostable mutants of a target enzyme.^17^ Among them, irrational design is a process of using random mutagenesis to obtain a stable protein including oligonucleotide-directed mutagenesis, error-prone PCR, and DNA shuffling.^18^ Based on the analysis of known structural and functional knowledge, semi-rational approaches identify amino acids that are more likely to yield positive results and mutate them by site-saturated mutagenesis.^17^ The algorithms of rational design are created based on studying available structure and sequence data of already known stable proteins. A new variant of the protein which is assumed to participate in stabilization by rational design methods is introduced by site-directed mutagenesis.^16^ Based on Rosetta computational protein design software in conjunction with rational design, Shirke *et al.* worked toward thermostabilization of *Aspergillus oryzae* cutinase, which led to a 6 °C improvement in *T*_m_ and a 10-fold increase in the half-life at 60 °C.^19^ Although all the three categories mentioned above can get good results, they differ significantly in the size of screening library. Comparison of protein stabilization methods by Bednar *et al.* revealed that significant advances towards rational approaches were seen to reduce the experimental effort required to engineer highly stable proteins.^20^ The experimental efforts for screening were >10 000 in irrational design procedures, 1000–10 000 using semi-rational protocols, and usually <100 *via* rational design methods. In view of this comparison, we assumed that rational design of stable mutants to reduce experimental efforts may be suitable to improve thermal stability of Lip1.

Thus, in this study, putative stable mutants were identified with three prediction algorithms. Identified residues in regions of vital-function were discarded. The remaining mutants were expressed, purified, and characterized. Homology modelling was employed to investigate the structural basis for improved stability. The results indicated that a hot-spot with enhanced thermal stability was successfully identified using site-directed mutagenesis based on rational design methods.

## Materials and methods

### Materials

The Lip1 gene sequence was optimized and constructed based on the protein-coding sequence of *C. rugosa* Lip1 (GenBank accession number: X64703.1)^6^ (ESI DOC S1†). *Escherichia coli* DH5α was maintained at 37 °C in Luria-Bertani medium for gene cloning. The vector pPICZαA (Novagen, Darmstadt, Germany) and *Pichia pastoris* GS115 were used for gene expression. All *P. pastoris* transformants were cultured in YPD medium (1% yeast extract, 2% tryptone, and 2% dextrose), BMGY medium (2% tryptone, 1% yeast extract, 50 mM potassium phosphate, pH 7.5, 1.34% yeast nitrogen base, 4 × 10^−5^% biotin, 1% glycerol) and BMMY medium (2% tryptone, 1% yeast extract, 50 mM potassium phosphate, pH 7.5, 1.34% yeast nitrogen base, 4 × 10^−5^% biotin, 1% methanol) containing 100 μg mL^−1^ zeocin (Invitrogen, Carlsbad, CA, USA) at 28 °C. Restriction endonucleases, Taq DNA polymerase, DNA Ligation Kits and PrimeSTAR™ HS DNA Polymerase were purchased from TaKaRa (Otsu, Japan). Kits for plasmid extraction, and gel extraction were bought from Omega Bio-tek (Norcross, GA, USA). *p*-Nitrophenyl (*p*-NP) esters were commercially obtained from Sigma-Aldrich (USA). All other chemicals were got from Sino-pharm Chemical Reagent Co., Ltd (Shanghai, China).

### Rational prediction of potential thermostable mutants

The crystal structure of Lip1 (PDB ID: 1CRL) was used as the template for thermostable mutant prediction. FoldX, Rosetta ddg_monomer and I-Mutant 3.0 were all used to identify the thermostable mutants for characterization.

FoldX is a method that evaluates protein stability by decomposing the Gibbs energy of protein folding into protein energy terms using empirical data from protein engineering experiments.^21^ When using FoldX for prediction, the 〈RepairPDB〉 module of FoldX suite (http://foldxsuite.crg.eu accessed 12 October 2017) was used for protein repair. The output was processed by the 〈BuildModel〉 module to estimate mutation effects. Settings were temperature 313 K, pH 8.0, and ion strength 0.05 M. Effects of mutations were determined by averaging five calculations (overall script is shown in ESI DOC S2†).

The Rosetta ddg_monomer program predicts mutation-induced stability changes with limited backbone relaxation. Change in stability is obtained as the difference in Rosetta energy between wild-type and mutant.^22^ The Rosetta ddg_monomer application was also used (http://www.rosettacommons.org accessed 12 October 2017). PDB files were processed with Rosetta pre_minimization script to obtain minimal energy confirmations. The Rosetta ddg_monomer application was used to calculate Rosetta energy of mutants for comparison to the original protein (pre-minimized and prediction scripts are shown in ESI DOC S3†).

I-Mutant 3.0 (http://gpcr2.biocomp.unibo.it/cgi/predictors/I-Mutant3.0/I-Mutant3.0.cgi, accessed 12 October 2017) is a supporting vector machine-based predictor trained with a dataset derived from the Protherm database.^23^ I-Mutant 3.0 derives stability change upon single point mutation from either sequence or structural information. The result is calculated from the unfolded Gibbs free energy value of the mutated protein minus the value of the wild-type.^24^ An 1CRL code was entered into the PDB code box. The numbers of residues mutated were inserted into the Position box, with one-letter codes for mutants in the New Residue box. Temperature and pH were set at 40 °C and 8.0.

### Screening by conservation analysis and visual inspection

Evolutionary conservation profiles for Lip1 were available from ConSurf-DB (http://bental.tau.ac.il/new_ConSurfDB/ accessed 12 October 2017).^25^ Conservation scores were divided into a discrete scale of nine grades for visualization, from the most variable position (grade 1) to the most conserved position (grade 9). Residues with conservation scores ≥8 were regarded as highly conserved and not mutated further.

Residues within 5 Å of the catalytic triad have demonstrable effects on catalytic activity.^26^ These residues were selected by PyMOL^27^ and were not mutated. Residues in the active site, substrate-binding pocket, and lid region were also identified as functionally important and remained immutable.

### Site-directed mutagenesis

The Lip1 gene was inserted between *EcoR* I and *Not* I restriction sites to create the recombinant plasmid pPICZαA-Lip1 for site-directed mutagenesis. Polymerase chain reaction (PCR) was with PrimeSTAR™ HS polymerase (TaKaRa) and a temperature program of 98 °C for 5 min, 30 cycles of 30 s at 98 °C, 30 s at 68 °C, 1.5 min at 72 °C; and 5 min extension at 72 °C. PCR products were digested with *Dpn* I to remove parent plasmid and purified with PCR purification kits (Omega Bio-tek). Resulting DNA mixtures were transformed into *E. coli* DH5α competent cells. Cell suspensions were plated on Low Luria-Bertani (LLB) agar (1% tryptone, 0.5% yeast extract, 0.5% NaCl, 1.5% agar) containing zeocin (25 μg mL^−1^) and incubated at 37 °C until colonies appeared. All transformants from LLB-plates were washed with sterile water and pooled. Plasmids were extracted with plasmid DNA preparation kits (Axygen, Union City, CA, USA), linearized with *Sac* I, and transformed into *P. pastoris* GS115 competent cells by electroporation. Transformed *P. pastoris* cells were cultured on YPD plates containing zeocin 100 μg mL^−1^ at 28 °C for 3 days.

### Protein expression and purification

Single colonies were picked and inoculated in 50 mL BMGY. Recombinant cells were harvested by centrifugation at 4000 × *g* and transferred into 50 mL BMMY after incubation at 28 °C for 24 hours. In addition, 1% (v/v) methanol was added to cultures every 24 h to induce lipase expression. Culture supernatants were harvested by centrifugation at 4000 × *g* for 20 min, and concentrated by ultrafiltration using 10 kDa cut-off membranes (Millipore, Billerica, MA, USA). Crude enzyme solutions were dialyzed against NTA-0 buffer (50 mM Tris–HCl, pH 8.0, 500 mM NaCl) and loaded onto a Ni-affinity chromatography column. Proteins were washed with NTA-0, NTA-30 (50 mM Tris–HCl, pH 8.0, 500 mM NaCl, 30 mM imidazole), NTA-60 (50 mM Tris–HCl, pH 8.0, 500 mM NaCl, 60 mM imidazole) buffer, eluted with NTA-100 (50 mM Tris–HCl, pH 8.0, 500 mM NaCl, 100 mM imidazole) and NTA-200 (50 mM Tris–HCl, pH 8.0, 500 mM NaCl, 200 mM imidazole) buffer and dialyzed against 50 mM, pH 8.0 phosphate buffer to eliminate imidazole. Purified protein samples were verified using 12% sodium dodecyl sulfate polyacrylamide gel electrophoresis (SDS-PAGE). For western blots, proteins were transferred from SDS-PAGE onto polyvinylidene fluoride membranes. Membranes were blocked with 5% milk and incubated with anti-His tag (1 : 2000) primary antibody and horseradish peroxidase-conjugated goat anti-mouse IgG (Tiangen, Beijing, China). Blots were processed with diaminobenzidine.^28^

### Thermostability profiles

Differential scanning fluorimetry^29^ was carried out to determine apparent melting temperatures (*T*^app^_m_) of enzyme mutants. For each analyzed sample, 5 μL of 100× SYPRO^®^ Orange solution (Life Technologies, Carlsbad, CA, USA) and 20 μL purified protein (0.2 mg mL^−1^) were mixed and centrifuged at 13 400 × *g* for 5 min at 4 °C. *T*^app^_m_ was defined as maximal relative fluorescence change by temperature (dRFU/d*T*) by heating samples from 25 °C to 85 °C at 1 °C min^−1^ in a StepOnePlus™ Real-Time PCR System (Life Technologies, Carlsbad, CA, USA).

### Assays of enzyme properties

To determine substrate specificity, hydrolysis rates of *p*-NP acetate (C2), *p*-NP butyrate (C4), *p*-NP octanoate (C8), *p*-NP decanoate (C10), *p*-NP dodecanoate (C12), *p*-NP myristate (C14), and *p*-NP palmitate (C16) were monitored in 1 mL reaction systems containing 960 μL Tris–HCl buffer (50 mM, pH 8.0), 10 μL *p*-NP ester (100 mM), and 20 μL pure ethanol with 10 μL diluted enzyme solution (1 mg mL^−1^).

Optimal pH was determined by incubating purified enzyme in the reaction system at different pH values: citrate–phosphate buffer (pH 6.0–6.5), Tris–HCl buffer (pH 7.0–8.5), or glycine–NaOH buffer (pH 9.0–10.0). The highest activity measured was considered 100%.

To determine optimum temperature, Lip1 and mutants were incubated at 5 °C intervals for 5 min starting at 25 °C and increasing to 60 °C in 1 mL reaction systems. All experiments were performed in triplicate.

### Assays of thermal stability and pH stability

Enzyme inactivation half-life was calculated by measurement of enzyme residual activity from 0 to 100 min at 50 °C. Samples were centrifuged at 13 400 × *g* for 2 min to eliminate aggregated protein.

The pH stability of each enzyme was determined by assaying their residual activity at the standard conditions mentioned above after preincubation at their optimum temperature for 10 min in the pH range 6.0–10.0 without substrate. The buffers for the different pHs were 50 mM citrate–phosphate buffer (pH 6.0–6.5), Tris–HCl buffer (pH 7.0–8.5), or glycine–NaOH buffer (pH 9.0–10.0), as mentioned above.

### Assays of catalytic efficiency and activity

The kinetic parameters (*k*_cat_, *K*_m_, and *k*_cat_/*K*_m_) of the wild-type Lip1 and the mutant were determined using different concentrations of *p*-NP C4 at pH 8.0 and their optimum temperature. The kinetic parameters were determined from Lineweaver–Burk plots using Microsoft Excel.

Protein concentration was determined using the Bradford method with bovine serum albumin as the standard.^30^ The lipase activity was determined by the alkali titration method using olive oil as a substrate.^6,31,32^ The 10 mL reaction mixture included 4 mL of substrate (25% (v/v) olive oil emulsified with a 2% (m/v) polyvinyl alcohol solution), 5 mL of 50 mM Tris–HCl buffer (pH 8.0), and 1 mL of properly diluted enzyme solution. The reaction system was incubated at appropriate temperature for 10 min with 150 rpm of shaking. Finally, 15 mL of cold acetone/ethanol (1 : 1, v/v) was added to terminate the reaction. The amount of liberated fatty acid was measured by titration with 50 mM NaOH using phenolphthalein as an indicator. Measurements were conducted in triplicates. One unit lipase activity (*U*) was defined as the amount of enzyme liberating 1 μmol fatty acid per minute.

### Molecular modeling and analysis

Mutant structures were modeled with the SWISS-MODEL server (http://swissmodel.expasy.org/ accessed 12 October 2017) using the crystal structure of Lip1 as a template.^33^ Molecular graphics were created with PyMOL.^27^ Intermolecular interactions were analyzed with the iMutants program of the iRDP web server (http://irdp.ncl.res.in/index.html accessed 12 October 2017).^34^ Default settings were used for analysis.

## Results and discussion

### Rational prediction of residues to improve enzyme thermostability

Computer-aided design methods analyze crystal structure, predict conformational stability of each amino acid residue, calculate Gibbs free energy change upon mutation, and identify mutants that may improve thermal stability.^35^ Compared with random mutagenesis and site-directed saturation mutagenesis, computational design of thermostable mutants is rapid and targeted.^12,20^ Typical computational methods used are: FoldX, I-Mutant 3.0, Rosetta ddg_monomer, PoPMuSiC, Eris and CUPSAT.^36^ These algorithms are evaluated and compared by correlating coefficients, systematic bias, and self-consistency. I-Mutant 3.0 (structural mode) and FoldX were the most reliable predictors in a systematic analysis of 11 online stability predictors using 1784 single mutations in 80 proteins.^37^ Rosetta ddg_monomer had the best performance based on systematic bias and random errors compared with the other three methods.^38^ Based on recent reviews, three methods, Rosetta ddg_monomer, FoldX, and I-Mutant 3.0, are the most promising algorithms.^37,38^ With these forms of prediction, systematic and random errors are inevitable in single algorithm. Hence, combining the results from several prediction methods are more likely to reduce the possibility of false positives. Therefore, in this study, the three methods described above were intersected to estimate substitution effects on the enzymatic thermostability of Lip1.

Rosetta ddg_monomer and FoldX return negative values for stabilizing mutations and positive values for destabilizing mutants. 77 stable mutants were predicted by FoldX, 165 stable mutants were selected by Rosetta ddg_monomer, and 25 mutants were predicted by both algorithms. For these 25 mutants, the change of unfolding Gibbs free energy upon mutation (ΔΔ*G*^Unfold^) was further predicted using the I-Mutant 3.0 web server. Positive values predicted by I-Mutant 3.0 indicate improved stability whereas the negative values indicate destabilization. Then, six mutants, including Glu66Phe and 5 mutants of Asp457, were confirmed by all three rational design methods, which meant that these mutants were most likely to have a potential positive effect on protein stability (Table 1). This feature markedly reduced the effort required for further characterization.

**Table 1:** Stable mutants identified by all three computational methods and correspondent estimated value

Mutants	FoldX^a^ (kcal mol^−1^)	Rosetta ddg_monomer^a^ (kcal mol^−1^)	I-Mutant 3.0^b^ (kcal mol^−1^)
Asp457Phe	−6.08	−12.41	0.09
Asp457Leu	−4.73	−4.71	0.47
Asp457Met	−4.95	−5.15	0.15
Asp457Tyr	−4.99	−11.96	0.07
Asp457Trp	−4.21	−10.03	0.18
Glu66Phe	−2.63	−3.07	0.32

a Rosetta ddg_monomer returns negative value for stabilizing mutations. The greater the absolute value of negative numbers, the greater likelyhood to be more stable.

b Positive values predicted by I-Mutant 3.0 indicate that induced mutations are more stable.

### Screening for mutagenesis by conservation analysis and visual inspection

**Fig. 1:**
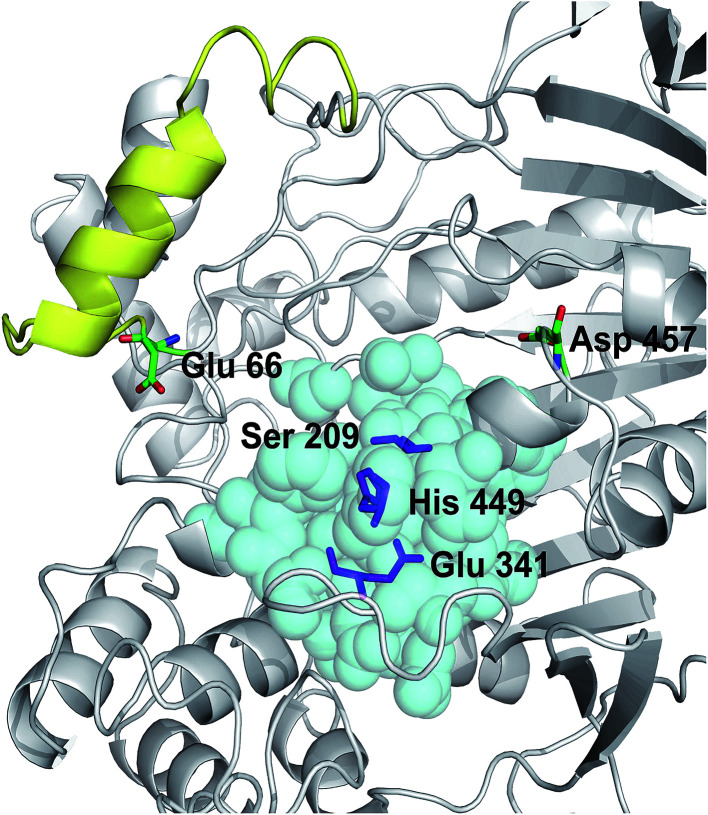
Positions of mutated residues. Catalytic triad, magenta sticks; residues around 5 Å of the catalytic triad, cyan balls; lid structure, yellow. Positions of Glu66 and Asp457 are also shown.

The crystal structure of Lip1 is resolved and the structural features of the catalytic triad, substrate binding pocket and other functional parts of Lip1 have been described.^39^ Lip1 contains 534 amino acid and is an α/β serine hydrolase with a typical active center composed of a catalytic triad, Ser209-Glu341-His449, and an α-helix “lid” structure that covers the top of the catalytic triad.^40^ These fundamental structures are important for maintaining the catalytic properties and stability of Lip1. Residues within 5 Å of the catalytic triad are often considered immutable for protein stability engineering.^12^ In addition, the selection of non-conserved residue sites as potential targets is due to the fact that these non-conserved residues are susceptible to changes in thermal stability during evolution.^41^ In view of these points, we analyzed and eliminated conserved-region residues likely to interfere with catalysis or substrate binding. The conservation grade for each amino acid residue of Lip1 was obtained from Consurf-DB. Conservation scores of Glu66 and Asp457 were 4 and 1, indicating that the two residues were not conserved. The position of mutated residues is in Fig. 1. Glu66 is located at the hinge region of the lid structure.^39^ Since catalysis of lipases is closely related to lid structure switching, mutations in the lid structure's hinge region were likely to result in alteration or loss of catalytic properties, such as enzymatic activity or enantioselectivity.^42,43^ Hence, Glu66 was not mutated. The Lip1 crystal structure showed that residue Asp457 was located at the loop region of α-helix 451 to 456 and α-helix 464 to 477, 5 Å away from the catalytic triad. We hypothesized that mutation of Asp457 would increase enzyme stability without compromising catalytic activity. Using three computational algorithms and visual inspection of important residues, five mutants of Asp457 (Asp457Phe, Asp457Leu, Asp457Met, Asp457Trp, and Asp457Tyr) were characterized for protein thermal stability.

### Enzyme expression and purification

**Fig. 2:**
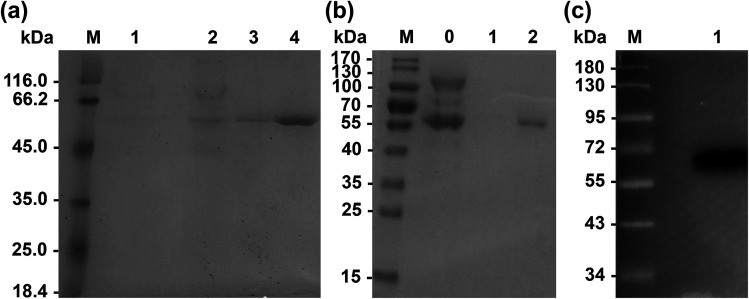
Expression of wild-type and recombinant Lip1. (a) SDS-PAGE of purified wild-type Lip1. M: protein marker; 1: fermentation liquid; 2: fermentation liquid after concentration; 3: eluate of NTA-100; 4: eluate of NTA-200. (b) SDS-PAGE of purified Asp457Phe. M: protein marker; 0: the fermentation liquid; 1: eluate of NTA-100; 2: eluate of NTA-200. (c) Western blot of Asp457Phe.

Primers used for mutations are listed in Table 2. Wild-type Lip1 and five mutants were expressed in *P. pastoris*. Supernatants were collected, concentrated after fermentation, and purified using Ni-affinity chromatography. Expression and purification of all constructed mutants resulted in good yields and protein purity. A SDS-PAGE image of the representative purified enzymes is shown. Single bands near 60 kDa were seen with SDS-PAGE and western blots, coinciding with the theoretical molecular weight of Lip1 (Fig. 2). Recovery of the target protein was better with NTA-200 buffer than with NTA-100, indicating that the protein was tightly bound to the nickel column. When the purification process is finished, 1.6 g purified wild-type enzyme was yielded per L of culture while 1.215 g purified Asp457Phe was obtained from 1 L of the culture. Then, the purified enzyme solutions were dialyzed in sodium phosphate buffer and diluted to 0.2 g L^−1^ in order to determine the enzyme properties.

**Table 2:** All primers used for mutation^a^

Primers	Sequence (5′–3′)	Size (bp)
Asp457Phe-F	gatcaaagaaccacttccaagcaagta**aaa**ctggaaaacaatatcgtttgagtgaaa	57
Asp457Phe-R	tttcactcaaacgatattgttttccag**ttt**tacttgcttggaagtggttctttgatc	57
Asp457Trp-F	caaagaaccacttccaagcaagta**cca**ctggaaaacaatatcgtttgagtg	51
Asp457Trp-R	cactcaaacgatattgttttccag**tgg**tacttgcttggaagtggttctttg	51
Asp457Tyr-F	gatcaaagaaccacttccaagcaagta**gta**ctggaaaacaatatcgtttgagtgaaa	57
Asp457Tyr-R	tttcactcaaacgatattgttttccag**tac**tacttgcttggaagtggttctttgatc	57
Asp457Met-F	gatcaaagaaccacttccaagcaagta**cat**ctggaaaacaatatcgtttgagtgaaa	57
Asp457Met-R	tttcactcaaacgatattgttttccag**atg**tacttgcttggaagtggttctttgatc	57
Asp457Leu-F	gatcaaagaaccacttccaagcaagta**aag**ctggaaaacaatatcgtttgagtgaaa	57
Asp457Leu-R	tttcactcaaacgatattgttttccag**ctt**tacttgcttggaagtggttctttgatc	57

a The forward and reverse primers for mutation were provided above. The codon for mutating Asp457 to other residues were shown as bold.

### Comparison of thermodynamic property between wild-type Lip1 and its mutants

**Fig. 3:**
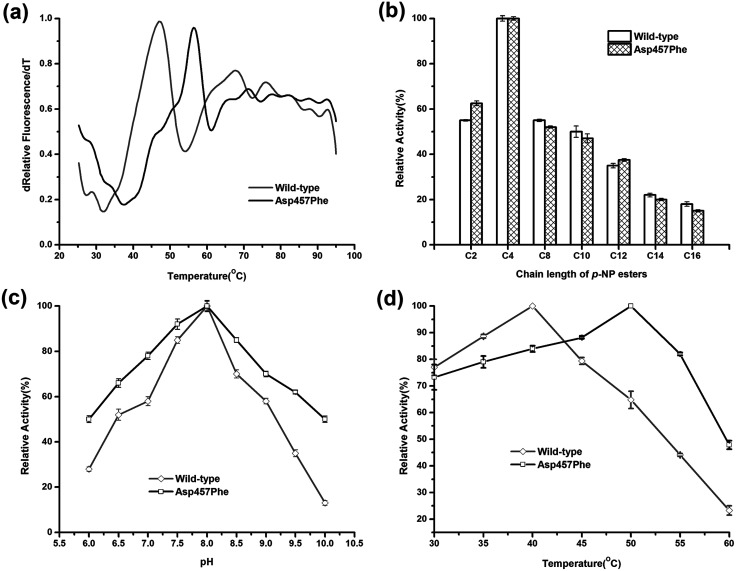
Comparison of enzymatic properties between wild-type Lip1 and Asp457Phe. (a) Normalized apparent melting temperature curve of wild-type Lip1 (gray) and Asp457Phe (black) detected using the DSF method. (b) Substrate specificity of wild-type (white) and recombinant Lip1 (filled). (c) Optimal pH of wild-type (gray) and mutated Lip1 (black). (d) Comparison of optimum temperature between wild-type (gray) and recombinant Lip1 (black).

Enzymatic thermodynamic stability is defined as the difference between the Gibbs free energies of the folded and unfolded conformations of the protein. To evaluate the change in enzyme thermodynamic stability as a result of mutation, apparent melting temperature (*T*^app^_m_, the temperature at which half of the protein was unfolded) was assessed for the purified proteins. In many cases, techniques such as differential scanning calorimetry (DSC), differential scanning fluorimetry (DSF), circular dichroism (CD) spectroscopy may be used to determine the change of melting temperature caused by mutation.^16^ Compared to other methods, the DSF experiment requires only small amount (10–25 μL) of protein with minimum concentration (0.1–0.3 mg mL^−1^). A 96-well plate inserted into real-time PCR machine allows the entire measurement process to be completed in less than 2 hours. These features make the use of DSF method more suitable for rapid measurement of protein thermal stability^44^. *T*^app^_m_ for wild-type Lip1 was 47.1 °C. *T*^app^_m_ was increased with substitutions of Asp457 by 8.4 °C with Leu, 7.4 °C with Met, 8.9 °C with Trp and 8.4 °C with Tyr (ESI Fig. S1†). Mutation of Asp 457 to Phe largely stabilized Lip1, resulting in a 9.3 °C increase in *T*^app^_m_ (Fig. 3a and Table 3). Thus, we identified five mutants that significantly improved thermal stability in a hot-spot, where the most thermostable mutant, Asp457Phe, was selected for further analysis.

**Table 3:** Comparison of enzymatic and kinetic parameters between wild-type Lip1 and Asp457Phe

Enzyme	*T* ^app^_m_ (°C)	*t*_1/2_ (50 °C) (min)	*T*_opt_ (°C)	*K*_m_ a (μM)	*k*_cat_ a (s^−1^)	*k*_cat_/*K*_m_ (s^−1^ μM^−1^)
WT	47.1	4.2	40	8.5 ± 0.8	4006 ± 127	473 ± 30
Asp457Phe	56.4	27.3	50	9.2 ± 0.7	4234 ± 151	461 ± 19

a Kinetic parameters of all enzymes were determined at their optimal temperatures.

### Comparison of the enzymatic properties between the wild-type enzyme and Asp457Phe

To explore the effects of Asp457Phe substitution on substrate specificity, different chain lengths of *p*-NP esters, from C2 to C16, were used. Both the wild-type enzyme and Asp457Phe preferred to hydrolyze short chain *p*-NP esters, with *p*-NP butyrate (C4) as the favored substrate (Fig. 3b).

The pH preference was determined using *p*-NP butyrate as hydrolysis substrate. Relative activity of both wild-type and mutated lipase peaked at pH 8.0. However, we found that the residual activity of Asp457Phe was higher than that of wild-type enzyme at acidic and alkaline conditions. Residual activities of Asp457Phe at pH 6.0 and 10.0 were both about 50% of its optimal acitivity while the wild-type decreased to 28% and 13% of its maximum activity, respectively (Fig. 3c).

On the other hand, the optimal temperature of the wild-type Lip1 was 40 °C, while Asp457Phe reached its highest activity at 50 °C, which was 10 °C higher than the wild-type. The catalytic activity of the wild-type enzyme at 50 °C decreased to about 65% of its optimal activity at 40 °C (Fig. 3d).

### Thermal and pH stability assays

**Fig. 4:**
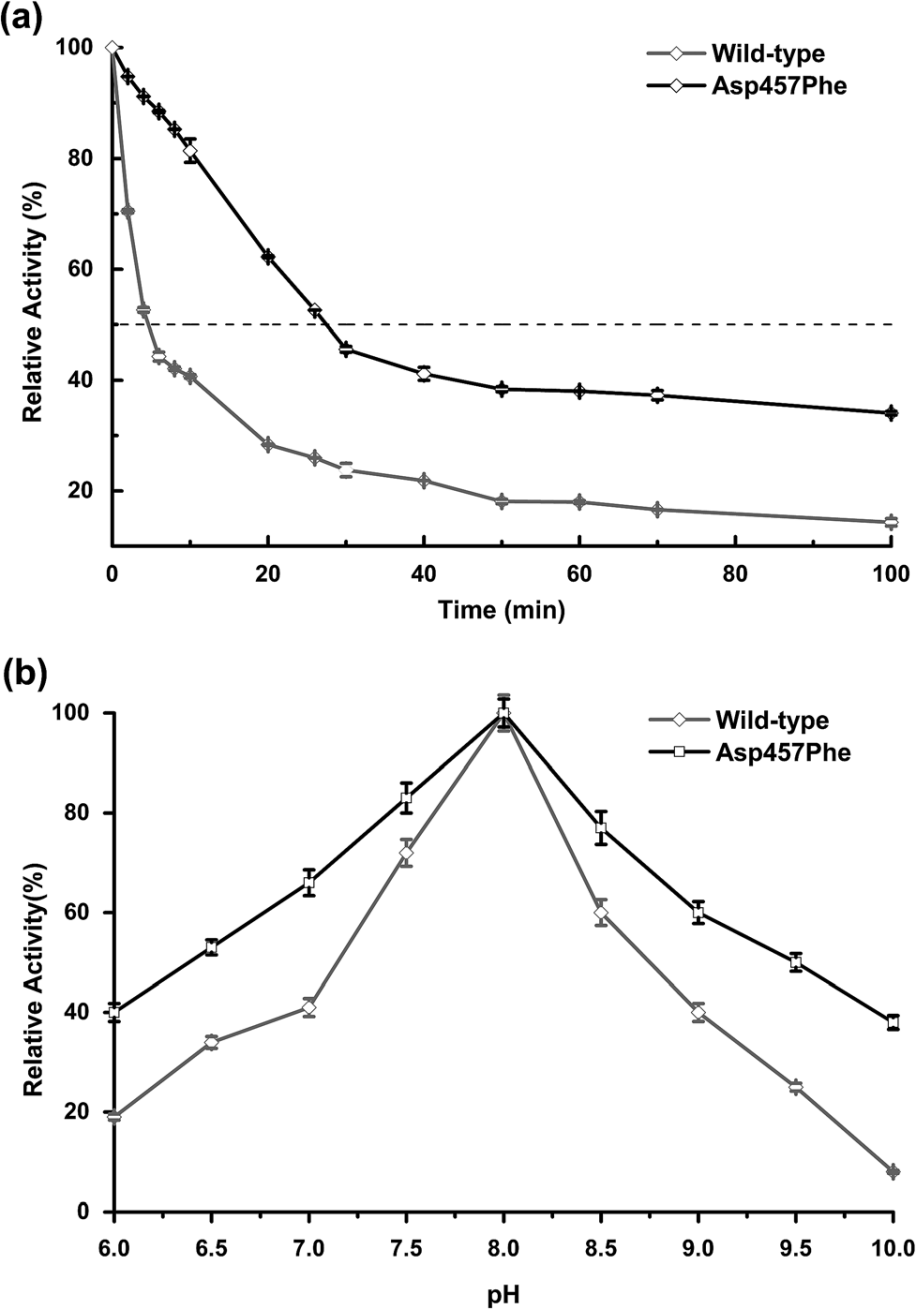
Comparison of thermal and pH stability between wild-type Lip1 and Asp457Phe. (a) Half-life of wild-type Lip1 (gray) and the mutant (black). (b) Residual activity of wild-type enzyme and the mutant after 10 min preincubation in different pH solutions.

Enzyme kinetic stability is defined as the time that protein remains functional before undergoing irreversible denaturation. The half-life (time for activity to be reduced by half at a specific temperature) is a parameter used to characterize changes in kinetic stability upon mutation.^15^ The half-lives of the wild-type Lip1 and Asp457Phe were assessed at 50 °C. The enzyme activity of wild-type Lip1 decreased sharply at the beginning of temperature elevation to a half-life of 4.2 min. The activity of the mutant protein decreased slowly at that temperature. The half-life of Asp457Phe at 50 °C was 27.3 min, which was 5.5-fold longer compared to that of the wild-type enzyme (Fig. 4a and Table 3).

In the process of testing the optimum pH, we found that the residual enzyme activity of the mutant was higher than that of the wild type at different pH conditions, so the pH stability of the wild type and the mutant was further determined. Asp457Phe showed improved stability after pre-incubation at 50 °C for 10 min in alkaline conditions (pH 7.0–10.0), and the stability of Asp457Phe increased by over 4-fold compared to that of the wild type in the extremely alkaline condition (pH 10.0). Furthermore, the mutant Asp457Phe had better resistance to acidic conditions (pH 6.0–7.0) (Fig. 4b).

### Catalytic efficiency and activity assays

The kinetic parameters of both enzymes were determined with *p*-NP C4 as the substrate at their own optimum temperatures and pH (Table 3 and ESI Fig. S2†). The *K*_m_ of Asp457Phe increased by 8.2%, indicating that substrate affinity of the mutant was lower than that of the wild-type Lip1. The catalytic constant (*k*_cat_) of Asp457Phe increased by 5.6%. Thus, the catalytic efficiency (*k*_cat_/*K*_m_) of Asp457Phe decreased by 2.6%.

The Asp457Phe mutation does not affect the structure of the Lip1 active site, allowing the mutant enzyme to completely retain its functional role. Phenylalanine is an aromatic and highly hydrophobic residue. It could involve in π–π stacking and π–cation interactions for maintaining conformational stability in protein structure.^45,46^ For both protein thermostability and substrate binding affinity, almost perfect enthalpy–entropy compensation is observed.^47^ Due to Asp457Phe mutation, the catalytic constant (*k*_cat_) was increased with a penalty to substrate binding affinity (increased *K*_m_). It appears to be caused by an increased entropic penalization to the enthalpically driven substrate-binding process.^48,49^ This phenomenon was also found in other engineered enzymes^50–52^

**Fig. 5:**
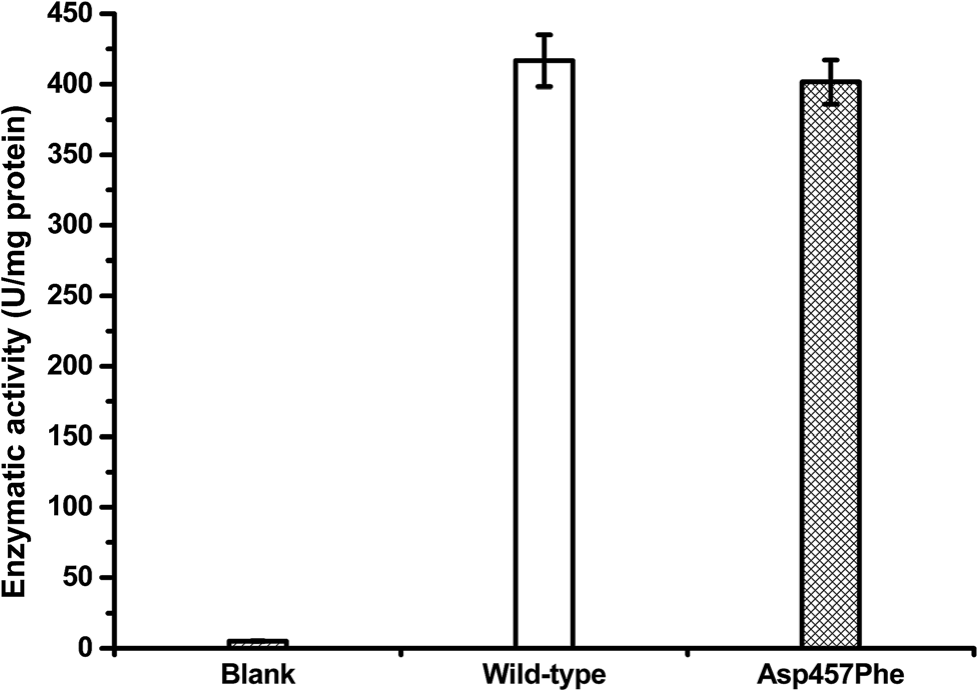
Lipase activity of wild-type Lip1 at 40 °C (white) and Asp457Phe at 50 °C (filled).

Protein concentrations were determined using the Bradford method,^30^ with wild-type protein 0.228 mg mL^−1^ and Asp457Phe 0.193 mg mL^−1^. Alkaline titration assays with olive oil emulsion were used to investigate lipase activity. When 1 mL of diluted enzyme solution was used for assay, lipase activity of Lip1 was 95 U at 40 °C, while that of Asp457Phe was 77.5 U at 50 °C. Hence, the specific activity of Lip1 at 40 °C was 416.67 U mg^−1^, and Asp457Phe was 401.55 U mg^−1^ at 50 °C (Fig. 5). Therefore, from the determination of catalytic efficiency and activity, the Asp457Phe mutation showed no significant catalytic difference from the wild-type Lip1.

### Analysis of increased thermal stability *via* mutation

To investigate the mechanism by which Asp457Phe and other mutants improved Lip1 thermal stability, three-dimensional models of the wild-type and all mutants were constructed by SWISS-MODEL. The quality of each constructed model was assessed using both the PROCHECK tool^53^ and the Verify3D server.^54^ The Ramachandran plot that evaluated stereochemical properties between residues in the model showed that 98.9% residues of Asp457Phe was in the allowed region, 0.4% in the generously allowed region, and only 0.7% in the disallowed region (ESI Fig. S3†). In addition, analysis of the Verify3D server showed a reliable folding in 85.58% of the residues in Asp457Phe model. In general, the constructed model fulfilled the requirements for further structure-based analysis. Inspection of the model structure of the Lip1 mutant revealed that mutation site 457 was located in a loop region on the surface of Lip1, which was related to the fact that the loop regions and surface areas are usually targeted to improve the thermal stability of the protein^55^ (Fig. 1). This strategy has been successfully applied to enhance the thermal stability of *Bacillus subtilis* subtilisin E and *B. subtilis* LipA.^55,56^ Intermolecular interactions were analyzed by the iMutants application of the iRDP web server.^34^ The program is designed to identify molecular interactions, such as hydrogen bonds, hydrophobic interactions, and disulfide bonds, which are well known to be the major structural factors that are responsible for protein thermal stability.

**Fig. 6:**
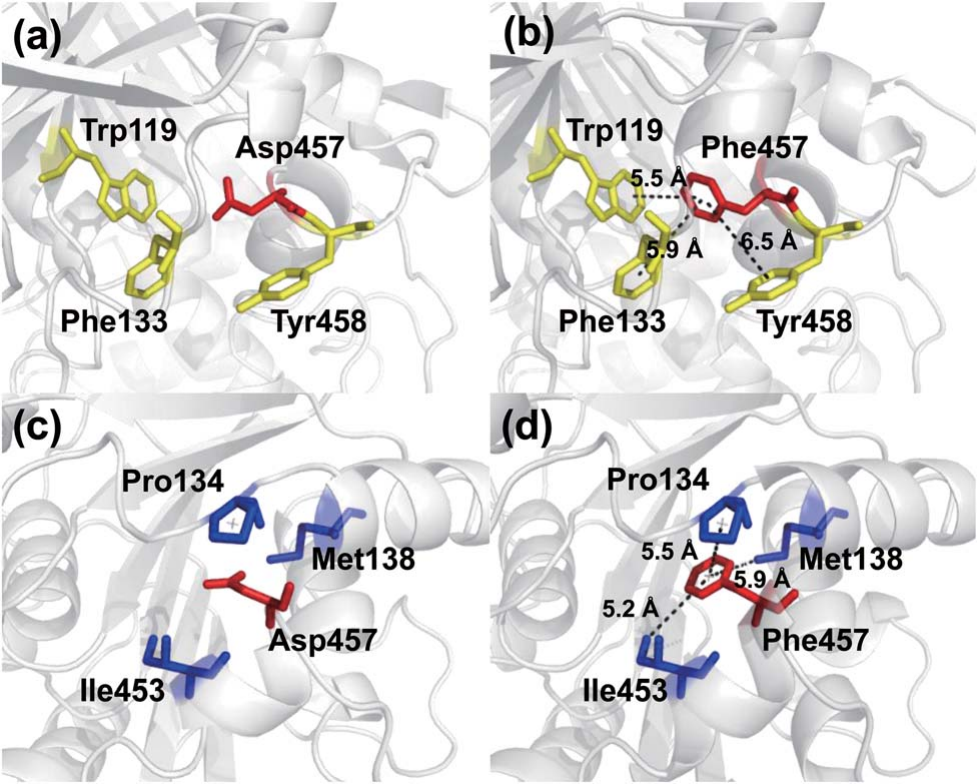
Comparison of intermolecular reactions of wild-type Lip1 and Asp457Phe. (a) Position of Asp457, red sticks; adjacent aromatic residues, yellow sticks. (b) Aromatic interactions between Phe457 (red) and adjacent residues (yellow). (c) Hydrophobic residues (blue) close to Asp457 (red). (d) Hydrophobic network including Phe457 (red) and other residues (blue).

The Asp457Phe model showed that the introduced phenylalanine created a putative aromatic–aromatic interaction network containing three residues (Trp119, Phe133, and Tyr458), thereby stabilizing the overall structure (Fig. 6a and b). Aromatic–aromatic interaction plays an important role in protein stability, and the introduction of aromatic–aromatic interaction can improve the thermal stability of the enzyme. Kannan *et al.* found that additional aromatic clusters or enlarged aromatic networks are present in many thermophilic protein families and absent in corresponding mesophilic homologs.^45^ That study highlighted aromatic clusters as a major contributing factor to protein stability. Furthermore, the intramolecular hydrophobic interactions of the wild-type and mutants were predicted in order to obtain insights into the possible structural effects on enzyme thermal stability. Substitution of Asp to Phe increased the hydrophobic interactions on the surface of the protein, which a hydrophobic network may be generated around Phe457 including Pro134, Met138, and Ile453 (Fig. 6c and d). Hydrophobic environments are important for thermophilic protein stability since most thermophilic proteins have higher hydrophobicity than their mesophilic counterparts. Compared with other factors, hydrophobic interactions contribute about 60% to protein stability.^57,58^ Increasing the number of hydrophobic interactions or generating a hydrophobic network is a common approach for protein stability engineering.^59^ In addition, enzyme inactivation under acidic and alkaline condition may be caused by the instability of hydrophilic residues on the molecular surface.^60^ Thus, increasing the hydrophobic interactions can regulate the pH tolerance of the protein, which is also consistent with the results of our experiments (Fig. 4b).

Similarly, an aromatic–aromatic interaction network (with Trp119, Phe133 and Phe206) and new hydrophobic interactions (with Pro134, Met138 and Ile453) may have formed with the introduction of Asp457Trp (ESI Fig. S4†). Substitution of hydrophilic Asp457 to hydrophobic Met formed a new hydrophobic network including Trp119, Phe133, Pro134 and Ile453 (ESI Fig. S5a and b†). Hydrophobic interactions between Phe133, Pro134, Ile453, Leu457 and Tyr458 mainly contributed to the improved stability of Asp457Leu (ESI Fig. S5c and d†). A hydrophobic network in which Tyr457 interacted with Pro134, Ala136 and Tyr458 was a key factor for enhancing the Lip1 thermal stability (ESI Fig. S5e and f†).

Based on the constructed model, more robust interactions may be formed in the hydrophobic network in Asp457Met, Asp457Leu, and Asp457Tyr than wild-type Lip1, since the substitution may lead to an upgrade in structural hierarchy of hydrophobic interaction clusters. In addition to the hydrophobic interactions, aromatic networks further improved the thermal stability of Asp457Phe and Asp457Trp. Combined with thermal stability measurement and model analyses, stabilization of Lip1 with hydrophobic and aromatic interactions was confirmed.

## Conclusion

Identification of putative mutants that enhance the thermal stability of eukaryotic proteins is challenging but important for academic and industrial applications. In this study, we found that five mutations at position 457 of Lip1 had a significant effect on enzyme thermal stability. These mutants were identified through a combination of three computational-prediction algorithms. Biochemical characterization revealed that the most thermostable mutant, Asp457Phe, had a 9.3 °C increase in apparent melting temperature, a 5.5-fold longer of half-life at 50 °C, retained more catalytic activity at the elevated temperature. Additionally, enhanced stability at acidic and alkaline conditions may also broaden practical applications of this CRL mutant. The study demonstrates that a hot-spot of thermally stable mutants in CRL can be identified with limited experimental efforts using rational design methods.

## Conflicts of interest

The authors declare that they have no conflict of interest.

## Funding

The authors acknowledge the financial support of National Natural Science Foundation of China (No. 31170078 and J1103514), National High Technology Research and Development Program of China (No. 2011AA02A204, 2013AA065805 and 2014AA093510), the National Natural Science Foundation of Hubei Province (No. 2015CFA085), and Fundamental Research Funds for Huazhong University of Science and Technology (No. 2014NY007, 2017KFTSZZ001 and 2017KFYXJJ212).

## Ethical approval

This article does not contain any studies with human participants or animals performed by any of the authors.

## Supplementary Material

RA-008-C7RA11679A-s001
