# Supplementary material for: Identification of a hot-spot to enhance Candida rugosa lipase thermostability by rational design methods
Source: RSC Adv. 2018 Jan 9;8(4):1948–57. doi: 10.1039/c7ra11679a (PMC9077275; doi:10.1039/c7ra11679a)
Supplement: RA-008-C7RA11679A-s001 [file RA-008-C7RA11679A-s001.pdf]

Electronic Supplementary Material (ESI) for RSC Advances.
This journal is © The Royal Society of Chemistry 2018

Supplementary materials

Identification of a hot–spot to enhance *Candida rugosa* lipase thermostability by rational design methods

Guanlin Li, Yuan Chen, Xingrong Fang, Feng Su, Li Xu*, Yunjun Yan*

Key Laboratory of Molecular Biophysics, the Ministry of Education; College of Life Science and Technology, Huazhong University of Science and Technology, Wuhan 430074, P. R. China

Corresponding Authors: Li Xu, Yunjun Yan

*Tel/Fax: +86-27-87792213

E-mail: xuli@hust.edu.cn; yanyunjun@hust.edu.cn

DOC S1 The codon-optimized gene sequence of wild-type Lip1

ATGGAGCTCGCTCTTGCGCTCTCTCTCATTGCCTCGGTGGCTGCTGCCCCT
ACTGCTACTCTTGCTAACGGTGACACTATCACAGGTCTTAACGCTATTATC
AACGAAGCCTTTTTGGGTATTCCTTTTGCCGAACCACCTGTTGGTAATCTT
AGATTCAAGGATCCAGTCCCTTACTCCGGATCATTGGACGGTCAAAAATT
CACTTCTTATGGACCATCTTGTATGCAACAGAACCCTGAGGGTACATACG
AAGAGAATTTGCCAAAGGCTGCCTTGGATCTTGTTATGCAGTCCAAAGTTT
TTGAAGCTGTCTCTCCTTCTTCCGAGGATTGCTTGACAATTAACGTTGTCA
GACCACCTGGAACCAAGGCCGGTGCAAATCTTCCAGTCATGTTGTGGATC
TTTGGTGGAGGTTTCGAAGTTGGAGGTACCTCTACTTTTCCACCTGCCCAA
ATGATTACTAAGTCCATCGCAATGGGTAAACCTATTATCCATGTTTCAGTC
AACTATAGAGTTTCAAGTTGGGGATTCTTGGCTGGAGATGAAATTAAGGC
CGAGGGATCTGCTAACGCCGGTCTTAAAGACCAAAGATTGGGTATGCAGT
GGGTTGCTGATAATATTGCAGCTTTTGGAGGTGACCCAACAAAGGTCACC
ATCTTCGGAGAGTCTGCTGGTAGTATGTCTGTTATGTGTCACATTTTGTGG
AACGATGGAGACAATACTTACAAGGGTAAACCATTGTTTAGAGCTGGAAT
CATGCAATCTGGTGCAATGGTTCCTTCTGATGCTGTTGATGGAATCTACGG
TAACGAAATCTTCGATTTGCTTGCTTCTAATGCCGGATGTGGTTCCGCCTC
AGACAAATTGGCATGCCTTAGAGGAGTTTCTTCCGATACATTGGAGGACG
CAACTAACAATACACCAGGTTTTCTTGCTTACTCAAGTTTGAGACTTTCTT
ATTTGCCAAGACCTGATGGTGTTAATATTACTGATGACATGTACGCTTTGG
TCAGAGAGGGAAAGTATGCCAACATCCCTGTTATTATCGGAGATCAGAAT
GACGAGGGAACTTTCTTTGGTACATCTTCTTTGAACGTCACTACAGATGCA
CAAGCTAGAGAATACTTTAAACAGTCTTTCGTTCATGCATCAGATGCTGA
GATTGACACCTTGATGACTGCTTATCCAGGAGATATTACACAAGGTTCTCC
TTTTGACACCGGTATCTTGAACGCCCTTACTCCACAGTTCAAAAGAATTTC
TGCAGTTTTGGGAGATCTTGGTTTTACTCTTGCTAGAAGATACTTCTTGAA
CCATTACACAGGAGGTACCAAGTACTCCTTCTTGTCTAAACAATTGTCTGG
ACTTCCTGTCTTGGGTACTTTTCACTCAAACGATATTGTTTTCCAGGACTA
CTTGCTTGGAAGTGGTTCTTTGATCTACAACAATGCCTTTATCGCCTTCGC

AACCGATTTGGACCCAAACACTGCTGGATTGCTTGTTAAGTGGCCTGAAT
ACACCTCAAGTTCTCAATCTGGTAACAATTTGATGATGATCAACGCTTTGG
GACTTTATACTGGTAAAGATAACTTTAGAACTGCTGGATACGACGCTCTTT
TTAGTAACCCACCTTCTTTCTTTGTCTAA

DOC S2 The running script of FoldX

```
<TITLE>FOLDX_runscript;
<JOBSTART>#;
<PDBS>1CRL.pdb;
<BATCH>#;
<COMMANDS>FOLDX_commandfile;
<RepairPDB>#;
<BuildModel>#,individual_list.txt;
<END>#;
<OPTIONS>FOLDX_optionfile;
<Temperature>313;
<R>#;
<pH>8;
<IonStrength>0.050;
<water>-CRYSTAL;
<metal>-CRYSTAL;
<VdWDesign>2;
<OutPDB>false;
<pdb_hydrogens>false;
<complex_with_DNA> true;
<moveNeighbours>true;
<numberOfRuns>5;
<END>#;
<JOBEND>#;
<ENDFILE>#;
```

DOC S3 The script for pre-minimization and prediction in Rosetta ddg_monomer algorithm

(a) Pre_minimization

```
/path/to/minimize_with_cst.linuxgccrelease
-in:file:l lst
-in:file:fullatom
-ignore_unrecognized_res
-fa_max_dis 9.0
-database /path/to/rosetta/main/database/
-ddg::harmonic_ca_tether 0.5
-score:weights standard_plus_score12
-ddg::constraint_weight 1.0
-ddg::out_pdb_prefix min_cst_0.5
-ddg::sc_min_only false
score:patch/rosetta/main/database/scoring/weights/score12.wts_patch>1CRL_A_mincst.log
```

(b) Prediction

```
-in:file:s 1CRL_A.pdb
-resfile 1CRL_A_1.resfile
-ddg:weight_file soft_rep_design
-database /patch rosetta /main/database/
-score:fa_max_dis 9.0
-ddg:iterations 50
-ddg:dump_pdbs true
-ignore_unrecognized_res
-ddg:local_opt_only true
-ddg:suppress_checkpointing true
-in:file:fullatom
-ddg:mean true
-ddg:min false
```

-mute all #

-ddg:ramp_repulsive false

-ddg:output_silent true

-ddg:opt_radius 8.0

-ddg:min_cst false

-ddg:sc_min_only false

-ddg:ramp_repulsive false

Table S1 Stable Mutants identified by FoldX[a]

Mutants	FoldX（kcal/mol）	Mutants	FoldX（kcal/mol）	Mutants	FoldX (kcal/mol)
**E341N**[b]	**-6.42**	N468M	-1.07	I253M	-0.35
**D457F**	**-6.08**	S527R	-1.04	N20R	-0.33
**D457Y**	**-4.99**	**A190W**	**-1.00**	S131Q	-0.30
**D457M**	**-4.95**	Y522F	-0.96	T493D	-0.29
**D457L**	**-4.73**	**S159G**	**-0.92**	**T130A**	**-0.28**
**G414W**	**-4.40**	V81K	-0.88	R279E	-0.26
**D457W**	**-4.21**	N528R	-0.85	P92R	-0.24
**S365M**	**-3.64**	**T13K**	**-0.82**	G383M	-0.22
**S365L**	**-3.44**	D10L	-0.78	G46C	-0.21
A22L	-2.70	Q137P	-0.75	L399M	-0.20
**E66F**	**-2.63**	**S531R**	**-0.72**	A398R	-0.18
**S365I**	**-2.57**	V323I	-0.69	N155T	-0.17
**A190F**	**-2.09**	Q187Y	-0.66	F391D	-0.16
**S93I**	**-1.91**	**E373W**	**-0.64**	T493H	-0.14
**T140M**	**-1.86**	**E257Y**	**-0.63**	A7H	-0.13
F391N	-1.73	S464M	-0.60	T427H	-0.12
N451L	-1.60	Q63L	-0.56	A4C	-0.11
**T476V**	**-1.59**	**D40W**	**-0.54**	L465M	-0.10
Q240M	-1.44	S495N	-0.51	D225H	-0.09
**A110P**	**-1.40**	Y361M	-0.50	E172G	-0.07
L478K	-1.37	T519R	-0.46	S270G	-0.06
A165P	-1.32	V189M	-0.44	N226V	-0.05
**E257W**	**-1.28**	G461M	-0.42	I219L	-0.04
H218L	-1.22	S272Y	-0.41	N265R	-0.03
W161P	-1.16	A263N	-0.39	N155S	-0.01
S495F	-1.12	K52L	-0.36		

[a] FoldX returns negative value for stabilizing mutations. The greater the absolute value, the greater likelyhood to be more stable.

b.Stable mutants identified by both Rosetta and FoldX were shown in bold.

Table S2 Stable Mutants identified by Rosetta ddg_monomer[a]

Mutants	Rosetta（kcal/mol）	Mutants	Rosetta（kcal/mol）	Mutants	Rosetta (kcal/mol)	Mutants	Rosetta（kcal/mol）	Mutants	Rosetta (kcal/mol)
**D457F**	**-12.412**	D340T	-2.558	S433A	-1.545	D318W	-0.941	**T130A**	**-0.209**
**D457Y**	**-11.955**	D340C	-2.524	I374L	-1.535	E71S	-0.932	D479T	-0.190
**D457W**	**-10.026**	G280K	-2.457	S84L	-1.524	**S93I**	**-0.913**	T379K	-0.164
D457H	-9.114	E70T	-2.438	E70N	-1.511	H425K	-0.888	S47Y	-0.145
**D457M**	**-5.513**	E21I	-2.419	E70C	-1.494	K147W	-0.886	V90A	-0.137
**E373W**	**-5.078**	D375T	-2.411	N256V	-1.479	E360T	-0.875	N101F	-0.129
**S365L**	**-5.008**	**E341N**	**-2.397**	K170Q	-1.453	D452E	-0.863	V33I	-0.108
**D457L**	**-4.706**	E491I	-2.336	D477H	-1.434	E325T	-0.843	T482Q	-0.092
**E257W**	**-4.315**	R405W	-2.280	**S531R**	**-1.411**	T379L	-0.820	S450C	-0.086
**S365M**	**-4.021**	T68C	-2.220	L478F	-1.391	L78M	-0.796	I374W	-0.064
**S365I**	**-3.952**	**A110P**	**-2.149**	E373H	-1.364	S464A	-0.788	R324K	-0.061
**S159G**	**-3.892**	K170Y	-2.052	D49L	-1.347	N192Y	-0.779	D96T	-0.054
S174W	-3.688	E491K	-2.011	**G414W**	**-1.322**	R303N	-0.751	K141T	-0.046
**A190W**	**-3.540**	G280Q	-1.987	**T476V**	**-1.315**	**T140M**	**-0.728**	K363V	-0.044
E126H	-3.389	D318H	-1.951	F434W	-1.300	N155H	-0.704	N72S	-0.039
**E257Y**	**-3.307**	D284V	-1.925	E21V	-1.280	Y69A	-0.671	A77T	-0.034
V534W	-3.288	K404L	-1.890	D375G	-1.267	L307Y	-0.645	A356T	-0.028
**D40W**	**-3.227**	Q182I	-1.842	D49W	-1.260	Q137W	-0.614	N351V	-0.025
**A190F**	**-3.214**	D49A	-1.818	A165W	-1.238	G129A	-0.589	K147Y	-0.023
**E66F**	**-3.074**	D248T	-1.794	E95I	-1.236	D371N	-0.566	S59H	-0.022
Q182L	-3.039	D284W	-1.773	Y432W	-1.217	D248G	-0.553	T493K	-0.021
K404M	-3.030	N72M	-1.771	Q83W	-1.191	N292W	-0.540	T227K	-0.017
D311N	-3.023	D375S	-1.759	D318V	-1.175	L465W	-0.524	D371W	-0.014
N468C	-2.936	E172A	-1.749	G129S	-1.145	D96A	-0.508	D355G	-0.010
E70L	-2.891	E360K	-1.730	D523C	-1.121	D49E	-0.488	N155Y	-0.010
V444T	-2.858	D318C	-1.709	L478Y	-1.103	**T13K**	**-0.469**	S370T	-0.009
E491V	-2.798	D167V	-1.685	K147H	-1.083	D167G	-0.450	V534N	-0.009
D318R	-2.745	N451S	-1.684	D284C	-1.073	I18A	-0.411	Y299V	-0.007
K431M	-2.682	D49Y	-1.665	K437W	-1.049	I19A	-0.374	E360S	-0.004
D412H	-2.664	L307F	-1.646	D318A	-1.039	D371S	-0.351	E88S	-0.002
A165T	-2.642	D384T	-1.614	D479S	-1.016	Q497A	-0.312	N256C	-0.002
G280W	-2.600	D40G	-1.590	D199N	-0.984	E257S	-0.251	T447N	-0.001
T68A	-2.560	G280A	-1.563	E71R	-0.954	D10K	-0.231	A507K	-0.001

[a] Rosetta ddg_monomer returns negative value for stabilizing mutations. The greater the absolute value, the greater likelyhood to be more stable.

b.Stable mutants identified by both Rosetta and FoldX were shown in bold.

Table S3 Stable Mutants identified by I-Mutant 3.0

Mutants	FoldX[a]（kcal/mol）	Rosetta ddg_monomer[a]（kcal/mol）	I-Mutant 3.0[b]（kcal/mol）
Thr13Lys	-0.82	-0.47	-0.72
Asp40Trp	-0.54	-3.23	-0.28
**Glu66Phe**	**-2.63**	**-3.07**	**0.32**
Ser93Ile	-1.91	-0.91	-0.25
Ala110Pro	-1.40	-2.15	-0.09
Thr130Ala	-0.28	-0.21	-1.73
Thr140Met	-1.40	-0.73	-0.44
Ser159Gly	-0.92	-3.89	-1.19
Ala190Phe	-2.09	-3.21	-0.38
Ala190Trp	-1.00	-3.54	-0.58
Glu257Trp	-1.28	-4.32	-0.22
Glu257Tyr	-0.63	-3.31	-0.29
Glu341Asn	-6.42	-2.40	-0.79
Ser365Ile	-2.57	-3.95	-0.24
Ser365Leu	-3.44	-5.01	-0.24
Ser365Met	-3.64	-4.02	-0.42
Glu373Trp	-0.64	-5.08	-0.23
Gly414Trp	-4.40	-1.32	-0.34
**Asp457Phe**	**-6.08**	**-12.41**	**0.09**
**Asp457Leu**	**-4.73**	**-4.71**	**0.47**
**Asp457Met**	**-4.95**	**-5.15**	**0.15**
**Asp457Tyr**	**-4.99**	**-11.96**	**0.07**
**Asp457Trp**	**-4.21**	**-10.03**	**0.18**
Thr476Val	-1.58	-1.31	-1.02
Ser531Arg	-0.72	-1.41	-0.66

[a] Rosetta ddg_monomer and FoldX return negative values for stabilizing mutations. The greater the absolute value, the greater likelyhood to be more stable.

[b] Positive values predicted by I-Mutant 3.0 indicate that induced mutations are stable.

Figure S1 *T*app m determination of CRL1 mutants

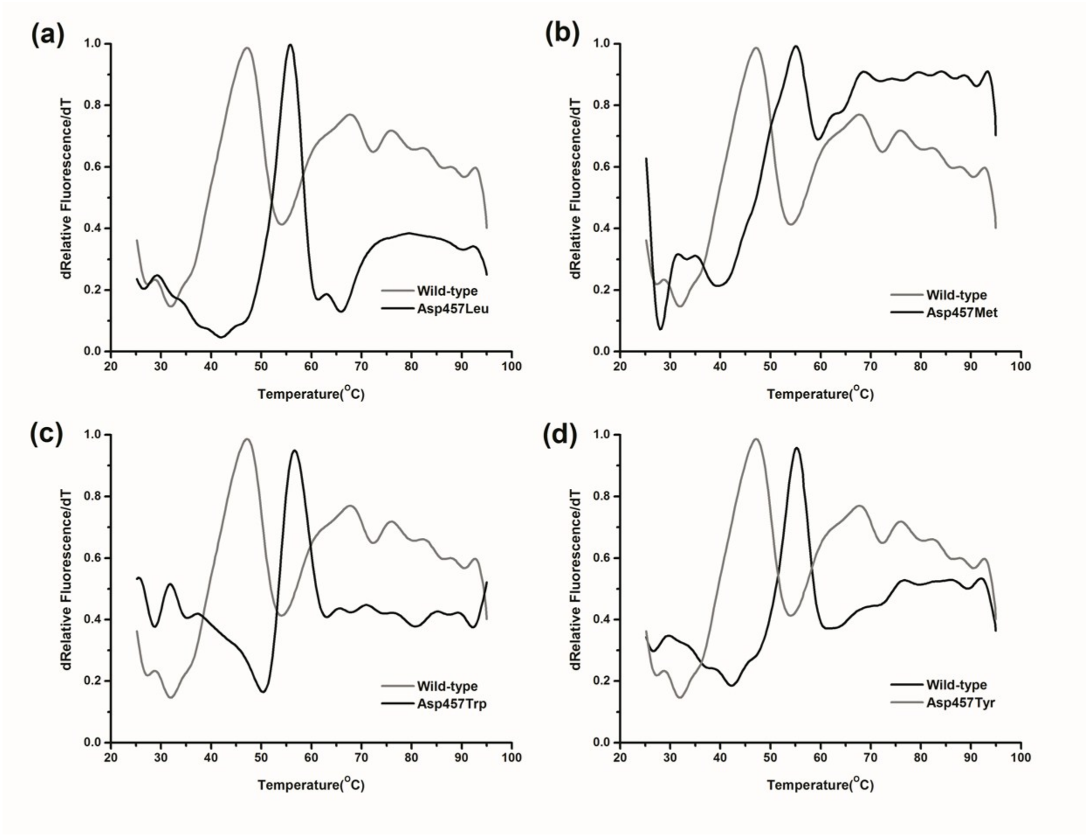

Comparison of normalized apparent melting temperature curve between wild-type Lip1 and mutants. **(a)** wild-type Lip1 (gray line) and Asp457Leu (black line), **(b)** wild-type Lip1 (gray line) and Asp457Met (black line) **(c)** wild-type Lip1 (gray line) and Asp457Trp (black line) **(d)** wild-type Lip1 (gray line) and Asp457Tyr (black line).

Figure S2 Substrate saturation curves of wild-type Lip1 and Asp457Phe

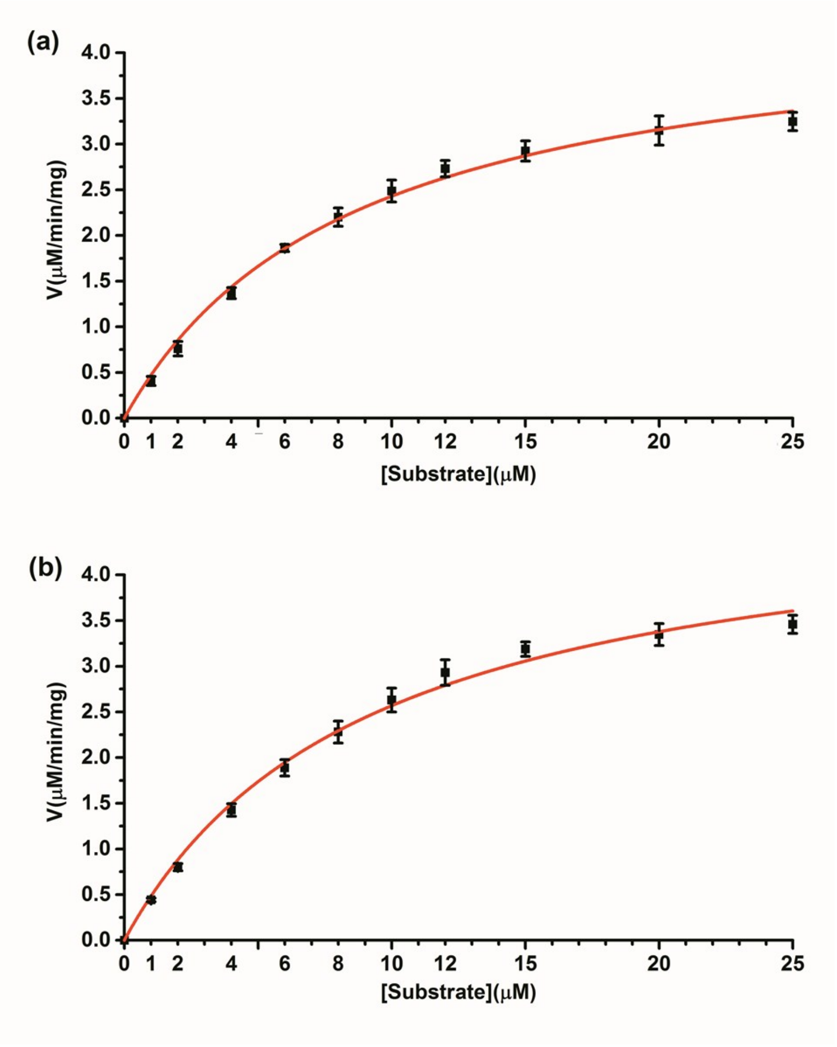

(a) substrate saturation curve of wild-type Lip1, **(b)** substrate saturation curve of Asp457Phe mutant.

Figure S3 Ramachandran Plot of Asp457Phe

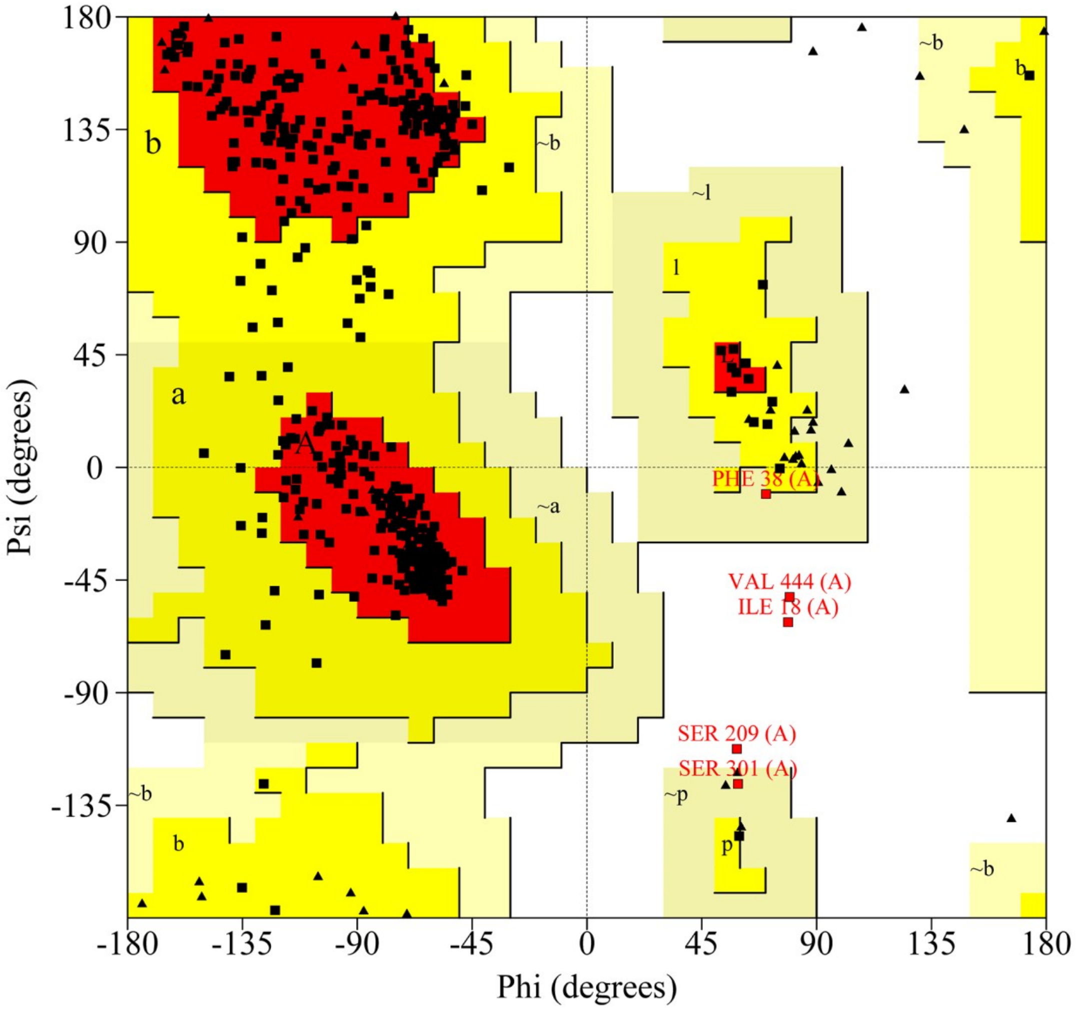

Plot statistics

Residues in most favoured regions [A,B,L]	391	87.9%
Residues in additional allowed regions [a,b,l,p]	49	11.0%
Residues in generously allowed regions [~a,~b,~l,~p]	2	0.4%
Residues in disallowed regions	3	0.7%
	----	------
Number of non-glycine and non-proline residues	445	100.0%
Number of end-residues (excl. Gly and Pro)	2	
Number of glycine residues (shown as triangles)	56	
Number of proline residues	31	

Total number of residues	534	

Figure S4 Comparison of intermolecular interactions in wild-type Lip1 and Asp457Trp.

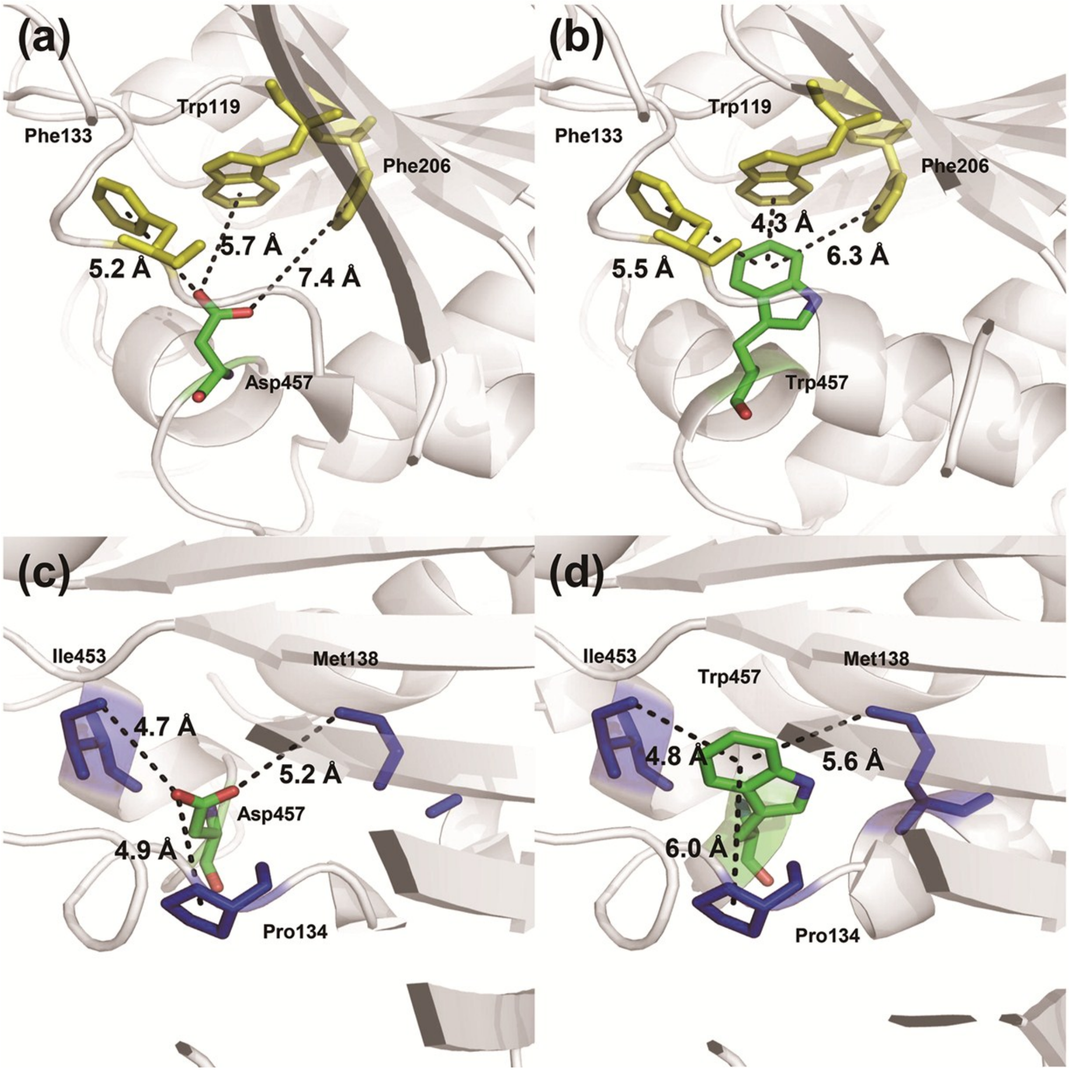

(a) The distance between Asp457 (colored by atoms) and aromatic residues nearby (yellow). **(b)** Trp457 (colored by atoms) induced aromatic interactions with Trp119, Phe133 and Phe206 (yellow). **(c)** The distance between Asp457 (colored by atoms) and adjacent hydrophobic residues (blue) **(d)** Trp457 (colored by atoms) induced hydrophobic interactions with Pro134, Met138 and Ile453 (blue).

Figure S5 Comparison of hydrophobic interactions in wild-type Lip1, Asp457Met, Asp457Leu, and Asp457Tyr.

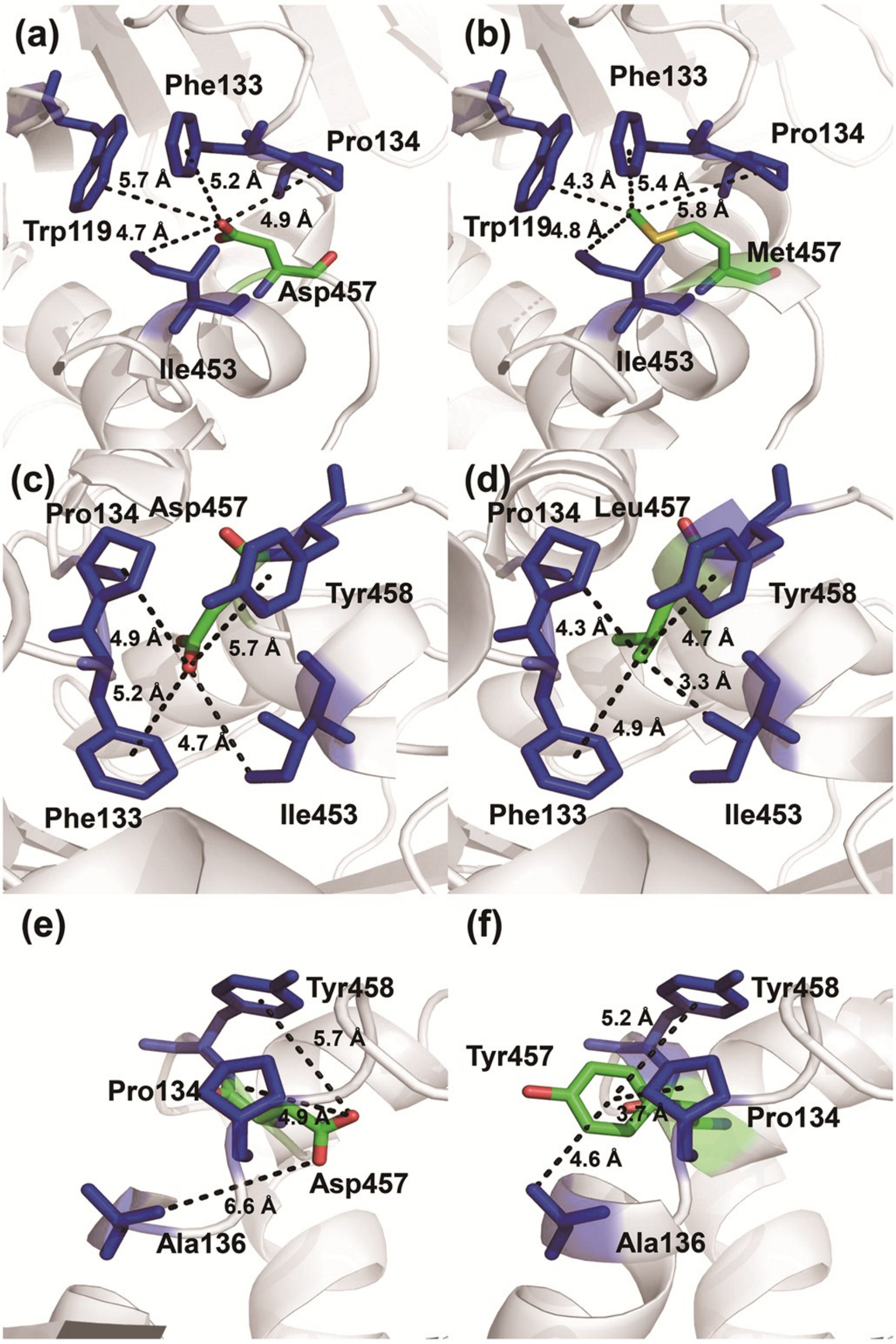

(a) The distance between Asp457 (colored by atoms) and adjacent hydrophobic residues (blue). **(b)** Met457 (colored by atoms) induced hydrophobic interactions with Trp119, Phe133, Pro134, and Ile453 (blue). **(c)** The distance between Asp457 (colored by atoms) and adjacent hydrophobic residues (blue). **(d)** Leu457 (colored by atoms) induced hydrophobic interactions with Phe133, Pro134, Ile453 and Tyr458 (blue). **(e)** The distance between Asp457 (colored by atoms) and adjacent hydrophobic residues (blue) **(f)** Tyr457 (colored by atoms) induced hydrophobic interactions with Pro134, Ala136 and Tyr458 (blue).
